# Photovoltaic Effect of La and Mn Co-Doped BiFeO_3_ Heterostructure with Charge Transport Layers

**DOI:** 10.3390/ma17092072

**Published:** 2024-04-28

**Authors:** Jiwei Lv, Huanpo Ning

**Affiliations:** College of Science, Donghua University, Shanghai 201620, China; 13085076925@163.com

**Keywords:** bismuth ferrite, doping, charge transport layers, photovoltaic

## Abstract

Bismuth ferrite BiFeO_3_ (BFO)-based ferroelectrics have great potential as inorganic perovskite-like oxides for future solar cells applications due to their unique physical properties. In this work, La and Mn co-doped BFO thin films with compositions Bi_0.9_La_0.1_(Fe_1−x_Mn_x_)O_3_ (x = 0, 0.05, 0.1, 0.15) (denoted as BLF, BLFM5, BLFM10, BLFM15, respectively) were prepared via a sol–gel technique on indium tin oxide (ITO) glass. All the films are monophasic, showing good crystallinity. The optical bandgap E_g_ was found to decrease monotonously with an increase in the Mn doping amount. Compared with other compositions, the BLFM5 sample exhibits a better crystallinity and less oxygen vacancies as indicated by XRD and XPS measurements, thereby achieving a better J–V performance. Based on BLFM5 as the light absorbing layer, the ITO/ZnO/BLFM5/Pt and ITO/ZnO/BLFM5/NiO/Pt heterostructure devices were designed and characterized. It was found that the introduction of the ZnO layer increases both the open circuit voltage (V_oc_) and the short circuit current density (J_sc_) with V_oc_ = 90.2 mV and J_sc_ = 6.90 μA/cm^2^ for the Pt/ BLFM5/ZnO/ITO device. However, the insertion of the NiO layer reduces both V_oc_ and J_sc_, which is attributed to the weakened built-in electric field at the NiO/BLFM5 interface.

## 1. Introduction

With the exacerbation of the global energy crisis, solar energy has attracted more and more attention, due to its cleanness and sustainability. The conventional pn junction semiconductor solar cells occupied the majority of the market share as they have high photoelectric conversion efficiency. By utilizing the light trapping photonic crystal patterns on the surface, their efficiency can even break the Lambertian limit~29% [1]. However, the fabrication and installation costs are generally high. In addition, the constraint of the Schockley–Queisser limit still hinders the single pn junction solar cell from converting more than 34% of the incident light’s energy [2]. In the case of ferroelectric photovoltaic (FEPV) effects, the photo-generated electron-hole pairs could be separated by the built-in electric field stemming from the remnant polarization due to the inversion symmetry breaking; therefore, the open circuit voltage (V_oc_) is not limited by their optical bandgaps [3]. Consequently, the power conversion efficiency of ferroelectric solar cells has the potential to surpass the Shockley–Queisser limit [4,5,6,7]. Compared with the metal halide perovskites (e.g., CH_3_NH_3_PbI_3_), the inorganic ferroelectric oxides have much higher environmental stability under harsh thermal, mechanical, and chemical conditions [3,4,5,6,7,8]. Moreover, the raw materials of ferroelectric solar cells are generally abundant and inexpensive.

Bismuth ferrite BiFeO_3_ (BFO) is a lead-free ferroelectric possessing rhombohedrally distorted perovsikte-like structure at room temperature. It exhibits many outstanding physical properties, such as a significant remnant polarization (~100 μC/cm^2^), room temperature multiferroicity, and a relatively small bandgap (~2.7 eV) lower than many other ferroelectrics, resulting in a relatively wide solar absorption spectrum [9,10]. Therefore, BFO-based materials have been arousing intense research interest for photovoltaic and optoelectronic applications since the last two decades. However, the photocurrent density of BFO film is much lower than its commercial counterparts, so its photoelectric conversion efficiency remains insufficient for practical applications. The photovoltaic performance of BFO has been improved by various means, such as bandgap engineering, ferroelectric domain engineering, interface engineering, electrode structure optimizing, and multi-layer structures, and so on [11,12]. Doping on the A-site with rare earth elements (such as La, Pr, and Ce) and the B-site with transition metal elements (such as Ti, Mn, and Cr) has been proven beneficial to reduce the optical bandgap and high leakage current of BFO [13,14]. Oxygen vacancies are found to be one of the dominating factors for the high leakage current density of BFO films [15]. Yan et al. reported that the La-doped BFO thin film showed a larger remanent polarization and decreased leakage current density due to the decrease in oxygen vacancies [16]. Tu et al. investigated the La-doped BFO film on the A-site, and the bandgap was lowered to about 2.22 eV, resulting from the reduction in oxygen vacancies and less microstructure defects [17]. Yang et al. reported that both the bandgap and leakage current density decreased in Mn-doped BFO thin films, owing to the factors of reduction in the density of oxygen vacancies, enlargement of the grain size, and stabilization of the perovskite-like structure [18]. Diliautas et al. found that single-phase BiFe_1−x_Mn_x_O_3_ samples could maintain up to 25% Mn substitution [19].

In perovskite solar cell devices, electron transport layers (ETL) and hole transport layers (HTL) play a vital part and they are devoted to efficiently transporting photo-generated charge carriers while effectively blocking opposite charges and preventing photo-generated electrons and holes recombining at the interface. Inorganic charger transport materials exhibited a much higher environmental stability over the organic ones, such as Spiro-OMeTAD and PEDOT: PSS, and so on [20,21]. ZnO is an n-type semiconductor with a large optical bandgap of around 3.3 eV, and it exhibits good chemical stability and higher electron mobility compared to other electron transport materials, such as SnO_2_ and TiO_2_ [22,23]. Fan et al. reported the photovoltaic performance of ITO/ZnO/BFO/Pt heterostructure film with J_sc_ of 340 μA/cm^2^ and the photoelectric conversion efficiency of up to 0.33% [23]. The significant increase in the photocurrent was owing to the large amount of electron-hole pairs produced from ZnO. Tiwari et al. reported that the BFO/ZnO heterojunction solar cell showed a V_oc_ of 632 mV and a photo-conversion efficiency of 3.98%, which were among the highest reported values for all oxide solar cells [24]. As a p-type transparent conducting oxide with a wide bandgap of 3.5–4.0 eV, NiO has excellent chemical stability and superior hole transporting ability, which can help in efficiently collecting holes and reducing the charge carrier recombination losses [25,26]. Renuka et al. fabricated a WS_2_/BFCrO/NiO heterojunction device with both electron and hole transport layers, and found that the V_oc_ and J_sc_ both increased compared to the device with just one single electron or hole transport layer, leading to an efficiency improvement of about 70% [25]. Although research on the improvement of the photovoltaic performance of BFO has made great progress, it still faces many challenges in practical applications to date, such as its larger bandgap compared to the commercially available solar cell materials, low photo-generated charge carrier transportability, and high leakage current density due to the high density of defect states, so the photoelectric conversion efficiency is still far behind that of the photovoltaic materials currently employed for large-scale applications [3].

In this study, we prepared La and Mn co-doped BFO thin films with the compositions Bi_0.9_La_0.1_(Fe_1−x_Mn_x_)O_3_ (x = 0, 0.05, 0.1, 0.15) (denoted as BLF, BLFM5, BLFM10, BLFM15, respectively) via the sol–gel method to investigate the effect of Mn doping on the photovoltaic performance of BFO-based solar cells. Moreover, in order to investigate the interface effect and achieve an effective separation and transportation of charge carriers, ZnO film was introduced in the device as an electron transport layer (ETL) and NiO film as a hole transport layer (HTL) to construct both the ITO/ZnO/BLFM5/Pt and ITO/ZnO/BLFM5/NiO/Pt heterostructure devices. By means of both the compositional and structural design, this research is aimed to improve and also gain a better understanding of the factors that affect the photovoltaic performance of the BFO-based thin films.

## 2. Materials and Methods

### 2.1. Solution Preparation

All the precursor solutions were prepared by the sol–gel route. To prepare the ZnO film, 0.01 mol of zinc acetate (Zn(CH_3_COO)_2_) was dissolved in 2-methoxyethanol, and the mixture solution was stirred for 2 h at room temperature. Subsequently, 0.01 mol of ethanolamine was added and stirred for 1 h. This resulted in a clear and transparent solution. For the BLFM layer, stoichiometric amounts of bismuth nitrate pentahydrate (Bi(NO_3_)_3_•5H_2_O), iron nitrate nonahydrate (Fe(NO_3_)_3_•9H_2_O), lanthanum nitrate hexahydrate (La(NO_3_)_3_•6H_2_O), and manganese acetate tetrahydrate (Mn(CH_3_COO)_2_•4H_2_O) were weighed and dissolved in a mixed solution of acetic acid and 2-methoxyethanol. After stirring for 3 h, an appropriate amount of acetylacetone was added as a chelating agent, and the solution was stirred for another 2 h. This results in a precursor solution with a concentration of 0.25 mol/L. The solution was then sealed and aged for 2 days at room temperature before use. For the NiO layer, the required amount of nickel acetate tetrahydrate (Ni(CH_3_COO)_2_•4H_2_O) was dissolved in 2-methoxyethanol and the mixture was stirred for 2 h at 60 °C. Ethanolamine was subsequently added in a 1:1 molar ratio with nickel acetate. After stirring for 2 h, a green and clear solution was obtained. The solution was cooled to room temperature and aged for 1 day before use.

### 2.2. Films’ Preparation

All the thin films were deposited by the spin-coating technique. The BLFM samples without charge transport layers were directly deposited on the ITO (indium tin oxide) glass substrate. Prior to spin-coating, the ITO substrate was ultrasonically cleaned in acetone, anhydrous ethanol, and deionized water for 10 min, respectively. It was then heated on a hot plate to remove the residual water. Afterwards, the BLFM precursor solution was spin-coated on ITO at a speed of 3000 r/min for 30 s and then dried on a 150 °C hot plate for 5 min. It was then annealed at 550 °C for 5 min in a rapid thermal annealing oven. The spin-coating, drying, and annealing process was repeated multiple times to achieve the desired thickness. To prepare the ZnO/BLFM5/NiO heterojunctions, the ZnO precursor solution was firstly spin-coated onto the ITO glass at a speed of 4000 r/min for 30 s. It was then dried at 200 °C on a hot plate for 10 min. The spin-coating and drying process was repeated for the required thickness, and finally the film was annealed at 550 °C for 1 h. Afterwards, the BLFM layer was spin-coated on the ZnO layer as described above. To prepare the NiO layer, the precursor solution was spin-coated onto the BLFM5 thin film at a speed of 3000 r/min for 30 s. Finally, the samples were annealed in an annealing furnace at 500 °C for 30 min.

### 2.3. Films’ Characterizations

The crystal structure and phase purity of the film samples were inspected using an X-ray diffractometer (Bruker, D8, Karlsruhe, Germany) with CuKα radiation. The surface and cross-sectional microstructures of all the films were observed with a Field Emission Scanning Electron Microscope (FE-SEM) (Hitachi, SU8010, Tokyo, Japan). The absorbance spectra of the samples were measured in the wavelength range of 200–1000 nm by a UV–VIS–NIR spectrophotometer (Shimadzu, UV-3600, Kyoto, Japan). The X-ray and ultraviolet photoelectron spectra were acquired by a photoelectron microprobe (Thermo Fisher Scientific, Escalab 250Xi, Waltham, MA, USA) with monochromatized AlKa-radiation at room temperature. The J–V characteristics of the samples were tested under both dark conditions and simulated solar illumination (100 mW/cm^2^) from a xenon lamp (BBZM-I, Xuancheng, China) by a source meter (Keithley, 2450, Mansfield, TX, USA). Circular Pt electrodes were sputtered on top of the samples through a screen mask with a 0.8 mm diameter by an ion sputtering equipment (KYKY, SBC-12, Beijing, China). 

## 3. Results and Discussions

**Figure 1:**
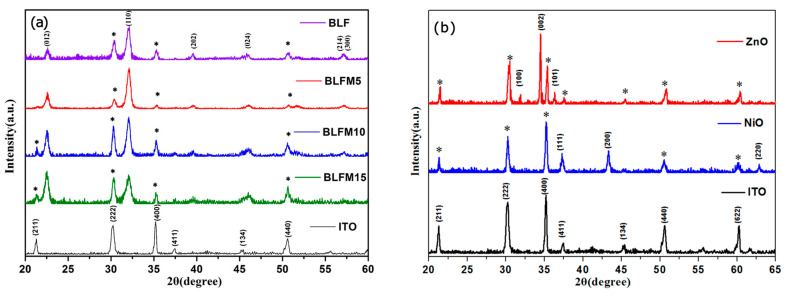
XRD patterns of (**a**) La and Mn co-doped BFO films spin-coated on ITO glass; (**b**) ZnO and NiO films spin-coated on ITO glass. The patterns of ITO glass substrate are also shown at the bottom and indexed with (*) in the patterns for all the coated films.

The XRD patterns of the La and Mn co-doped BFO films spin-coated on ITO glass are presented in Figure 1a. These patterns can be indexed as a rhombohedrally distorted perovskite having space group R3c, based on a standard PDF card (Number 86–1518). The peaks of the ITO glass substrate are also indexed (PDF: 65–3170) and denoted with (*) for all the deposited films. It can be seen that no discernible secondary impurity phase could be detected within the sensitivity limits of the XRD equipment, except for the peaks from the ITO substrate. Compared with other compositions, the BLFM5 sample exhibits much stronger BFO peaks compared with the ITO substrate, which indicates its better crystallinity. The average crystallite sizes of the La- and Mn-doped films were calculated according to Debye–Scherrer’s formula: D = Kλ/βcosθ, where D, K, λ, β, and θ stand for the crystallite size, the shape factor (0.9), the wavelength of the source X-ray (0.154 nm for CuKα radiation), the full width half maximum of the peak, and Bragg’s angle, respectively [27]. The XRD patterns of the ZnO and NiO films coated on the ITO glass substrate are shown in Figure 1b. It can be observed that the ZnO and NiO samples are both single phases showing good crystallinity, which can be indexed based on the standard PDF cards with number 75-0576 and 78-0429, respectively.

**Figure 2:**
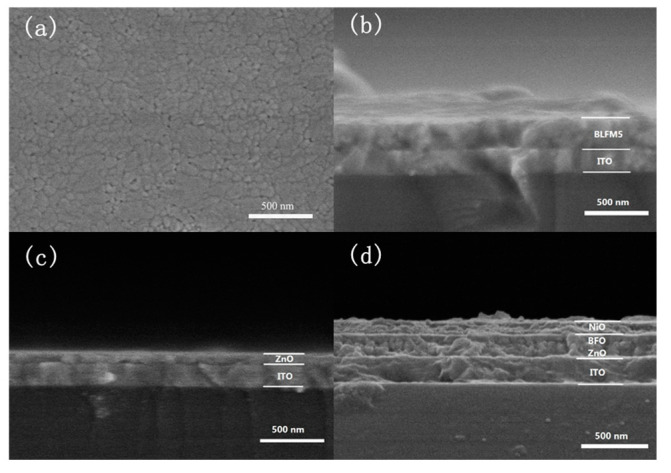
FE-SEM images of (**a**) surfaces of BLFM5 film; (**b**) cross-section of BLFM5 film coated on ITO glass; (**c**) cross-section of ZnO film coated on ITO glass; (**d**) cross-section of the ZnO/BLFM5/NiO heterostructure on ITO glass.

The microstructures of the surface and cross-sectional view of the spin-coated films are displayed in Figure 2a,b. From Figure 2a, it can be seen that the BLFM5 film exhibits a high density and polygonal grains, and only a few pores are observable at the grain boundaries. The distribution of grain size is not uniform, with the smaller grains having sizes ranging from 40 to 60 nm while the largest grains can have grain sizes above 200 nm. It can be observed from Figure 2b that the thickness of the BLFM5 film is uniform and around 200 nm, which is a little thicker than the ITO film with a thickness of about 180 nm. Figure 2c shows that the ZnO film coated on ITO glass is also uniform and the thickness is approximately 70 nm. The cross-sectional morphology of the heterostructure with the ZnO and NiO charge transport layers sandwiching the BLFM5 layer is shown in Figure 2d. The thickness of the top NiO layer is estimated to be around 100 nm and it also has a clear interface with the BLFM5 layer. However, the ZnO layer could not be clearly identified from the cross-sectional view after the depositing of the BLFM5 and NiO layers from Figure 2d. This could be due to the merging of the interface between ZnO and BLFM5, which is similar to the SEM result shown in Fan’s research [23].

**Figure 3:**
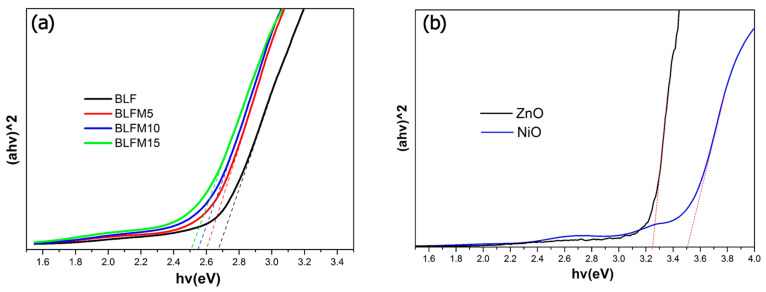
The optical bandgaps of (**a**) La and Mn co-doped BFO films; (**b**) ZnO and NiO films obtained from the (αhν)^2^ versus hν plot by the UV–Vis spectra measurement.

Figure 3 shows the optical bandgap *E_g_* of La and Mn co-doped BFO films, and also the ZnO and NiO films directly coated on ITO glass. The *E_g_* was determined by extrapolating the linear portion of the (αhν)^2^ vs. hν curves, in accordance with Tauc’s relationship [28]. It can be seen from Figure 3a that *E_g_* is about 2.66 eV, 2.60 eV, 2.55 eV, and 2.51 eV for BLF, BLFM5, BLFM10, and BLFM15, respectively. The bandgap monotonously decreases with the increasing Mn doping amount, which may be attributed to the gap states introduced between the conduction band and valence band [29]. The absorption within the bandgap could be due to the oxygen vacancies generated by the Mn doping. The *E_g_* was estimated to be 3.26 and 3.51 eV for the ZnO and NiO film, respectively, which is consistent with previous research results [22,26].

**Figure 4:**
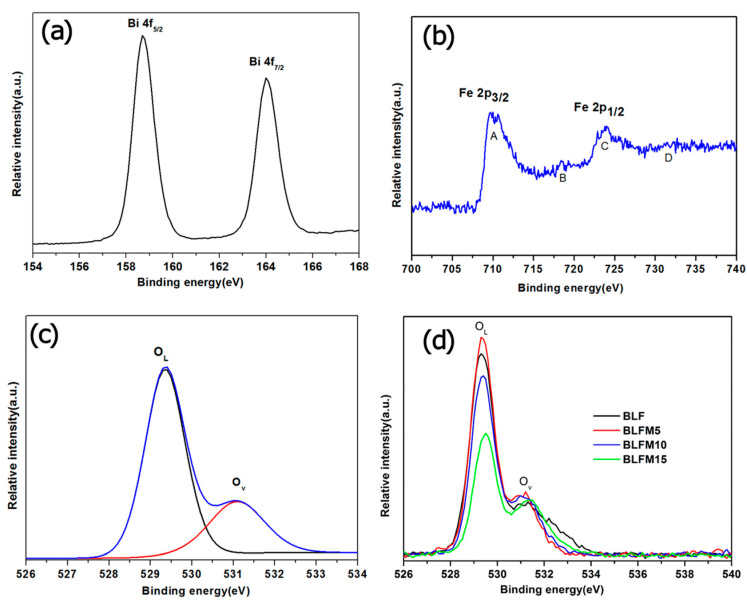
The core-level XPS spectra of the BLFM5 film. (**a**) Bi 4f, (**b**) Fe 2p, (**c**) O1s. (**d**) Comparison of the O1s spectrum for films with different Mn amount.

Figure 4a–c show the X-ray photoelectron spectra (XPS) spectra for Bi, Fe, and O for the BLFM5 film. The peak binding energy of Bi 4f_7/2_ and Bi 4f_5/2_ was 158.8 eV and 164.1 eV, respectively, which confirms the trivalent state of bismuth ions [26]. In Figure 4b, Fe 2p peak A and peak C reveal the Fe 2p_3/2_ and Fe 2p_1/2_ energy levels, respectively, which are attributed to the spin–orbit interaction. The energy separation between peak A and C is 13.3 eV. Peaks B and D represent the charge transfer satellites resulting from the screening of the initial Fe 2p vacancy by the electrons from neighboring atoms [30]. The satellite peak is around 718.4 eV, which is about 8 eV above the Fe 2p_3/2_ peak, indicating that Fe mainly exists in the trivalent state [30]. As can be seen from Figure 4c, the XPS spectra of O 1s have two peaks at around 529.4 eV and 531.1 eV, respectively. The former peak could arise from the intrinsic lattice oxygen ions O_L_ and the latter could be due to the adsorbed oxygen or oxygen vacancies O_v_ [25,26,27]. Figure 4d compares the O1s spectrum for BLFM films with a different Mn amount. It can be seen that the BLFM5 shows the highest O_L_ relative intensity peak compared with the peak of O_v_, which may imply the least oxygen vacancies existing in the sample. This improvement could be owing to the oxygen vacancy trapping effect of Mn dopant atoms, as reported by Nakashima [31]. However, when the Mn doping amount increases to 10% and 15%, the relative intensity peak decreases, which indicates an increase in oxygen vacancies. This could be attributed to a higher concentration of defects produced at the grain boundaries with the decrease in grain size and crystallinity [18,32].

**Figure 5:**
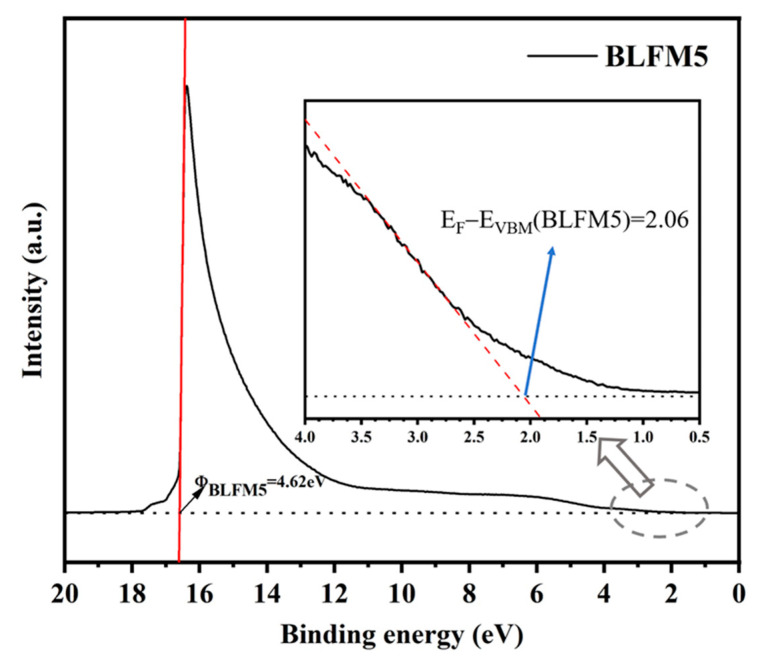
UPS spectra of the BLFM5 film with the inlet displays enlarged spectra focusing on the low binding energy edge.

Figure 5 shows the ultraviolet photoelectron spectra (UPS) of the BLFM5 film. After the bias correction, the work function of the BLFM5 film was calculated to be 4.62 eV by subtracting the spectral width from the photon energy (21.22 eV) of the excited radiation. The energy level of the E_F_−E_VBM_ (valence band maximum) value of BLFM5 was derived to be 2.06 eV by extrapolating the linear portion of the low binding energy edge to the position of the energy axis. Considering that the bandgap of BLFM5 obtained from the UV–Vis measurement in Figure 3a is 2.60 eV, the E_CBM_−E_F_ (conduction band minimum) can be calculated to be 0.54 eV. Therefore, BLFM5 can be regarded as an n-type semiconductor [23].

**Figure 6:**
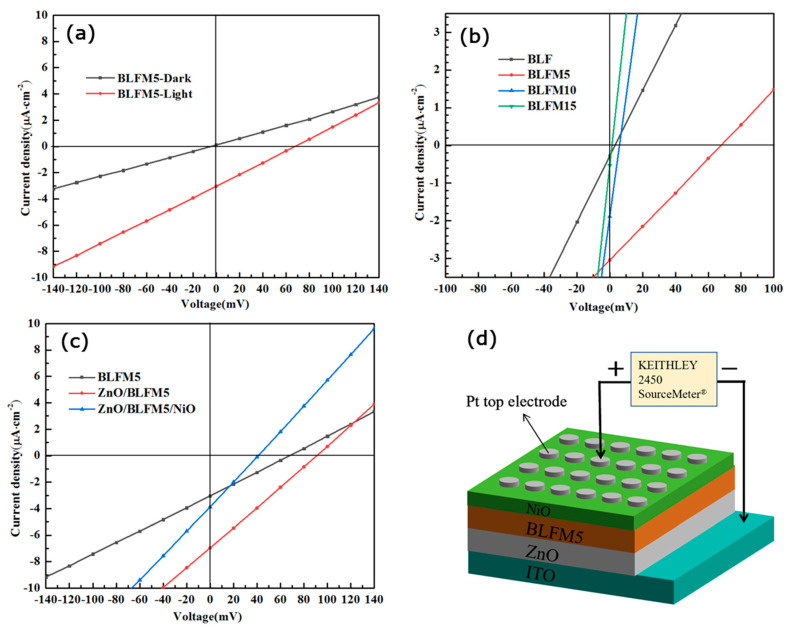
Current density–voltage characteristics of (**a**) ITO/BLFM5/Pt under dark and illuminated conditions; (**b**) different amounts of Mn-doped BLFM device under illuminated conditions; (**c**) BLFM5 device with just ZnO layer and with both ZnO and NiO layer under illuminated conditions. (**d**) A schematic of the ITO/ZnO/BLFM5/NiO/Pt heterostructure thin film for J–V measurement.

Figure 6 characterizes the current density–voltage (J–V) characteristics of the La- and Mn-doped BFO device with or without charge transport layers. Figure 6a displays the J–V characteristics of the ITO/BLFM5/Pt heterostructure under both illuminated and dark conditions. It can be observed that under dark conditions, there is no obvious photovoltaic effect. Under illuminated conditions, the open-circuit voltage and short-circuit current density can be determined from the intercepts with V_oc_ = 67.8 mV and J_sc_ = 3.04 μA/cm^2^. The negative photocurrent density indicates that the photo-generated holes move from the BLFM5 towards the Pt electrode under the electric field. As was discussed above, BLFM5 is an n-type semiconductor with a work function of 4.62 eV, which is lower than that of Pt (5.36 eV), so it can be inferred that the Schottky contact is formed at the Pt/BLFM5 interface with the direction of the built-in electric field E_bi-1_ pointing from BLFM5 to Pt. As for the BLFM5/ITO interface, considering that the work function of BLFM5 (4.62 eV) is greater than that of ITO, and BLFM5 is an n-type semiconductor, it can be deduced that an ohmic contact is formed at the BLFM5/ITO interface. Figure 6b shows the effect of Mn doping on the J–V performance of BLFM thin films without charge transport layers under illuminated conditions. Without the presence of Mn, the open-circuit voltage V_oc_ and short-circuit current density J_sc_ of BLF is only 3.2 mV and 0.28 μA/cm^2^, respectively. However, when the Mn doping amount reaches 5%, the V_oc_ is significantly enhanced to 67.8 mV, and the J_sc_ increases by one order of magnitude to 3.04 μA/cm^2^. The improvement in the photovoltaic effect of BLFM5 may be due to several reasons. Firstly, it could be attributed to the reduction in oxygen vacancies, as was discussed in Figure 4d. This is in agreement with Yang’s findings that for BFO thin films with 0–12% Mn doping, the optimum ferroelectric properties and lowest leakage current density were achieved in 8% Mn-doped BFO, owing to the stabilization of the perovskite structure, increase in the grain size, and reduction in the oxygen vacancies [18]. Secondly, larger polarization due to better crystallinity and a larger crystallite size could separate the electron and hole pairs more effectively [27]. Thirdly, a reduced bandgap compared with the BLF sample could allow it to absorb more photons in a wider solar spectrum. However, when the doping amount of Mn continues to increase to 10% and 15%, the V_oc_ and J_sc_ of the thin film both decrease sharply. This may be caused by the domination of the oxygen vacancies with an increase in the Mn amount, as shown in Figure 4d, which was reported to increase the recombination rate of photo-generated carriers [18]. Matsuo et al. reported that the local electric field across the domain walls of BFO films could be suppressed by Mn doping due to a screening effect by charged defects [33]. 

Due to the better J–V performance of the BLFM5 sample than other compositions, it was selected as the light absorbing layer in the heterojunction device with the charge transport layers. Figure 6c presents the J–V characteristics of the BLFM5 device with just the ZnO layer, and also with both the ZnO and NiO layers under illuminated conditions. The ITO/ BLFM5/Pt device exhibits V_oc_ = 67.8 mV and J_sc_ = 3.04 μA/cm^2^. After inserting the ZnO layer, the V_oc_ and J_sc_ of the ITO/ZnO/BLFM5/Pt device are significantly enhanced compared to the device without the ZnO layer, with V_oc_ reaching 90.2 mV and J_sc_ exceeding twice the previous value at 6.90 μA/cm^2^. This indicates that the high electron mobility of the ZnO layer indeed facilitates the migration and collection of the photo-generated electrons as expected. However, when the NiO layer was introduced, it can be observed that both the V_oc_ and J_sc_ of the ITO/ZnO/BLFM5/NiO/Pt device decrease evidently to approximately half the value of the device without NiO. Figure 6d shows the schematic of the ITO/ZnO/BLFM5/NiO/Pt heterostructure thin film device for the J–V measurement.

**Figure 7:**
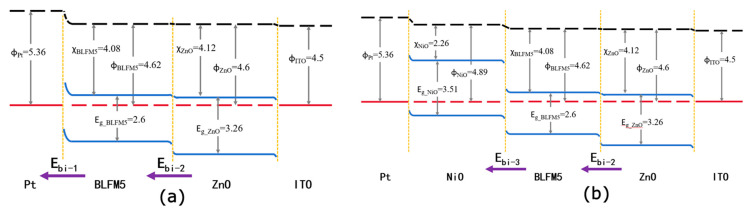
Energy band diagrams of (**a**) ITO/ZnO/BLFM5/Pt and (**b**) ITO/ZnO/BLFM5/NiO/Pt heterostructure.

Based on the UPS and optical bandgap data derived from Figure 3 and Figure 5, the energy band diagram of the ITO/ZnO/BLFM5/Pt heterostructure is illustrated in Figure 7a. Given that an ohmic contact forms at the BLFM5/ITO interface, and ZnO is an n^+^ type semiconductor, it may deduce that an n-n^+^ junction will generate at the BLFM5/ZnO interface in the ITO/ZnO/BLFM5/Pt structure [34,35], and there exists a built-in electric field E_bi-2_ pointing from ZnO to BFLM5 due to the interface potential difference. Meanwhile, the interface between ZnO and the ITO substrate forms an ohmic contact; therefore, the introduction of the ZnO layer could improve the movement of photo-generated carriers on the whole. However, when the NiO layer was introduced, it can be observed that both the V_oc_ and J_sc_ of the ITO/ZnO/BLFM5/NiO/Pt device decrease. It might be explained as follows. Since NiO is a p-type semiconductor with a work function of 4.89 eV [26], it is inferred that an ohmic contact and a Schottky contact are formed at the Pt/NiO and NiO/BLFM5 interfaces, respectively. As is shown in Figure 7b, a built-in electric field E_bi-3_ is produced at the NiO/BLFM5 interface pointing from BLFM5 to NiO. Considering the work function difference of Pt and NiO, however, this built-in field E_bi-3_ produced at the NiO/BLFM5 interface is smaller than the built-in electric field E_bi-1_ generated at the original Pt/BLFM5 interface without the NiO layer. Consequently, the net built-in field to separate photo-generated carriers is weakened and the photovoltaic effect is negatively influenced.

Table 1 summarizes the crystallite size, bandgap (E_g_), short circuit photocurrent density (J_sc_), and the open circuit photovoltage (V_oc_) of La and Mn co-doped BFO thin films. As is shown in Table 1, the crystallite sizes of La and Mn co-doped samples were calculated from Debye–Scherrer’s formula to be 16.3 nm, 27.7 nm, 16.8 nm, and 11.6 nm for BLF, BLFM5, BLFM10, and BLFM15, respectively. The BLFM5 sample clearly exhibits the largest crystallite size of all the samples, which corresponds to its best crystallinity shown in the XRD result in Figure 1a. This might suggest that a proper amount of Mn could promote the grain growth of BFO. However, when the Mn amount increases above 5%, the crystallite size gradually decreases. A similar trend of grain size with Mn doping was reported by Diliautas [19], and it was considered that the excessive addition of Mn could cause densification problems, thus restraining the grain growth. The larger crystallite size of BLFM5 may also account for its larger V_oc_ and J_sc_, since grain boundaries and defects could have the potential to function as recombination centers, capturing the photo-generated carriers [32].

## 4. Conclusions

In summary, the La and Mn co-doped BFO thin film heterostructure was prepared via the sol–gel technique on ITO glass. The optical bandgap E_g_ was found to decrease monotonously when the Mn doping amount increased from 0 to 15%. From the XRD and XPS results, the BLFM5 sample shows a better crystallinity and less oxygen vacancies than other samples with different Mn amounts. As a result, the BLFM5-based device shows a better photovoltaic performance with V_oc_ = 67.8 mV and J_sc_ = 3.04μA/cm^2^ under illuminated conditions. After introducing ZnO as the electron charge transport layer, V_oc_ and J_sc_ are significantly enhanced to 90.2 mV and 6.90 μA/cm^2^, which was attributed to the enhanced built-in electric field across the interfaces. However, the insertion of the NiO layer weakened the net built-in electric field, and reduced both V_oc_ and J_sc_ in the ITO/ZnO/BLFM5/NiO/Pt device.

## Figures and Tables

**Table 1:** Comparison of the crystallite sizes, optical bandgaps, V_oc_ and J_sc_ of the La and Mn co-doped BFO films.

Compositions	Crystallite Size (nm)	Optical Bandgap (E_g_) (eV)	V_oc_ (mV)	J_sc_ (μA/cm^2^)
BLF	16.3	2.66	3.2	0.28
BLFM5	27.7	2.60	67.8	3.04
BLFM10	16.8	2.55	5.9	1.88
BLFM15	11.6	2.51	1.4	0.53

## Data Availability

Dataset available on request from the authors.
